# Spread of Water-Borne Pollutants at Traffic Accidents on Roads

**DOI:** 10.1007/s11270-017-3477-3

**Published:** 2017-08-12

**Authors:** Bo Olofsson, Hedi Rasul, Annika Lundmark

**Affiliations:** 1 Division of Land and Water Resources Engineering, Royal Institute of Technology (KTH), 100 44 Stockholm, Sweden; 2 Water Unit, County Administration Board of Jämtland, 831 86 Östersund, Sweden

**Keywords:** Road, Pollution, Electrical resistivity, Infiltration, Modeling

## Abstract

Traffic accidents sometimes lead to the spread of hazardous compounds to the environment. Accidental spills of hazardous compounds on roads in the vicinity of vulnerable objects such as water supplies pose a serious threat to water quality and have to be assessed. This study compared three different assessment methods, electrical resistivity measurements, analytical flow calculations, and 1D and 2D dynamic flow modeling, to describe rapid transport processes in the road shoulder and roadside verge after a major spill. The infiltration and flow paths of water-borne substances were described during simulated discharge of pollutants on different road types. Full-scale tracer tests using sodium chloride were carried out at nine different road locations in Sweden. Analysis of grain size distribution and infiltrometer tests were carried out at the road shoulder and verges. The pathways and travel times were traced using resistivity measurements and 3D inverse modeling. The resistivity measurements were compared to analytical flow calculations and 1D and 2D dynamic modeling. All measurement sites were highly heterogeneous, which caused preferential flow. Vertical flow velocities of 1.4–8.6 × 10^−4^ m/s were measured. The results of the analytical calculations and flow modeling were of the same order of magnitude. The measurements showed that almost all infiltration goes directly into the road embankment, hence the composition and structure of the built-up road must be considered. The non-destructive resistivity measurements and 3D modeling provided useful information for clarifying the infiltration and flow pattern of water-borne compounds from road runoff.

## Introduction

Transport of hazardous liquids on roads sometimes results in accidents and discharge of pollutants. In Sweden, several thousand accidents are reported annually to the Swedish Rescue Services (Ohlén and Larsson 2000). The probability of traffic accidents causing water pollution can be estimated by Bayesian Network analysis (Yang et al. 2015; Tang et al. 2016). Liquid spills sometimes leave the paved road and infiltrate into the surroundings. Chemicals used to fight vehicle fires may also be more harmful to the environment than the fire itself (Källström and Mourujärvi 1999). Spread of pollutants from roads to surrounding surfaces and groundwater bodies has been studied frequently during recent decades (e.g., Thunqvist 2000, 2003; Lindström 2005; Moghadas et al. 2015; Aljazzar and Kocher 2016; Knez and Slabe 2016). Implementation of the EU Water Framework Directive (EG 2000/60) has increased awareness of the pollution risks along trafficked roads. Investigative monitoring under the Water Framework Directive is required to ascertain the magnitude and impacts of accidental pollution. In Sweden, several inventories have been made, focusing on areas where major roads cross important aquifers (Ojala and Mellqvist 2004; McCarthy et al. 2006). Methods for vulnerability assessment of pollution spread from roads have been developed (Rosén 1991; Gontier and Olofsson 2002), as well as methods for probability-consequence analyses (Rosén 1998). Comparable investigations have been made in Canada, Finland, and the United Kingdom (e.g., Soveri 1994; Nystén 1998; Rivett et al. 2016). The frequent use of de-icing salts during wintertime in the Scandinavian countries, Canada, and northern USA can be seen as a huge tracer experiment from roads to the surrounding environment. In Sweden, several studies have focused on the long-term spread of de-icing salts (Knutsson et al. 1998; Thunqvist 2003; Lundmark 2005, 2008). A GIS-based study using the national wells chemical archive at the Geological Survey of Sweden (SGU), combined with digital maps of the Swedish road network, indicated that as much as 50% of the chloride content in deep-drilled private wells within 500 m from major roads in Sweden originates from de-icing salt (Olofsson and Sandström 1998).

Most studies of pollution spread have focused on infiltration and percolation of the liquids in natural soils (e.g., Lundberg 1974; Lindblad 1981; Maxe and Johansson 1998), while only a few have examined flow paths in modern built-up roads. Risk analyses of pollution spread along drains and de-watering systems have been made by the Highways Agency (1996) in Scotland and by Benedetto and Cosentino (2003). Infiltration into the road at the edge of the asphalt layer has been studied by Hansson et al. (2005) and Stormont and Zhou (2005), who also point out the importance of unsaturated flow. Flow paths from the road surface have been modeled by Apul et al. (2007). Jensen (2004) conducted tracer experiments in sandy areas along four Danish highways and found that percolation was highly heterogeneous, with preferential flow along root systems and cables. The runoff and infiltration capacity of the vegetated inner slope of the road ditch has also been studied by Krarup et al. (1988), using single-ring infiltrometers. They found that infiltration rate was up to 10^−5^ m/s. An infiltration study along the highway E4 in Sweden using single-ring infiltrometers and Cornell infiltrometers, which simulated real precipitation situations, indicated that more than the total annual precipitation could infiltrate the road bank within an hour (Paulsson 2008).

However, knowledge of percolation processes in the road bank is still fairly limited, due to sampling and excavation problems on active roads. Instead of destructive drilling and chemical sampling, which is technically problematic and does not always give representative values in coarse heterogeneous road construction materials, indirect surface measurements, e.g., using geophysical methods, can be applied. Such methods have previously been used to study the development of groundwater pollution e.g., at industrial sites (Mao et al. 2015; Cuong et al. 2016) and from highly trafficked Swedish highways (Leroux and Dahlin 2005; Lundmark and Olofsson 2007; Olofsson and Lundmark 2009; Minas 2010; Earon et al. 2012). Comparable measurements using resistivity equipment to study the variation in water content in roads and ditch slopes have been carried out by Jackson et al. (2002). These studies focus primarily on natural infiltration. During traffic accidents, a large amount of pollutants may spread from paved surfaces. The main aim of the present study was to analyze the pathways of spread of water-borne pollutants from large road spills. A secondary aim was to develop and test a non-destructive method for tracing pollutant infiltration and percolation in various road environments due to a point discharge of polluted liquid. Water-soluble compounds which change the conductivity of the liquid were primarily used. An additional aim was to compare the tracer experiments with one-dimensional (1D) and two-dimensional (2D) unsaturated flow modeling and with an analytical solution for infiltration and percolation currently used by the Swedish Rescue Services.

## Methods

### Resistivity Measurements and Modeling

Tracing pollutants using geoelectrical methods is common practice in previous research (White 1994; French et al. 2002; Leroux and Dahlin 2005; Cassiani et al. 2006; Olofsson and Lundmark 2009). The general resistivity of the ground can be calculated as:

1 $$ \delta =\frac{V\times A}{I\times L} $$

where the resistivity (*δ*) is a function of the measured voltage (*V*), the current (*I*), and the cross-sectional area (*A*) of a conductor with length (*L*). The arrangement of the current and potential electrodes may vary depending on the aim of the geoelectrical study and the specific soil conditions. It can generally be written as:

2 $$ {\delta}_a=\frac{V}{I}\times \frac{2\pi }{\left[\frac{1}{\mathrm{AM}}-\frac{1}{MB}-\frac{1}{\mathrm{AN}}+\frac{1}{NB}\right]} $$

Where the measured apparent resistivity depends on the distance between the current electrodes (A, B) and the potential electrodes (M, N).

**Fig. 1:**
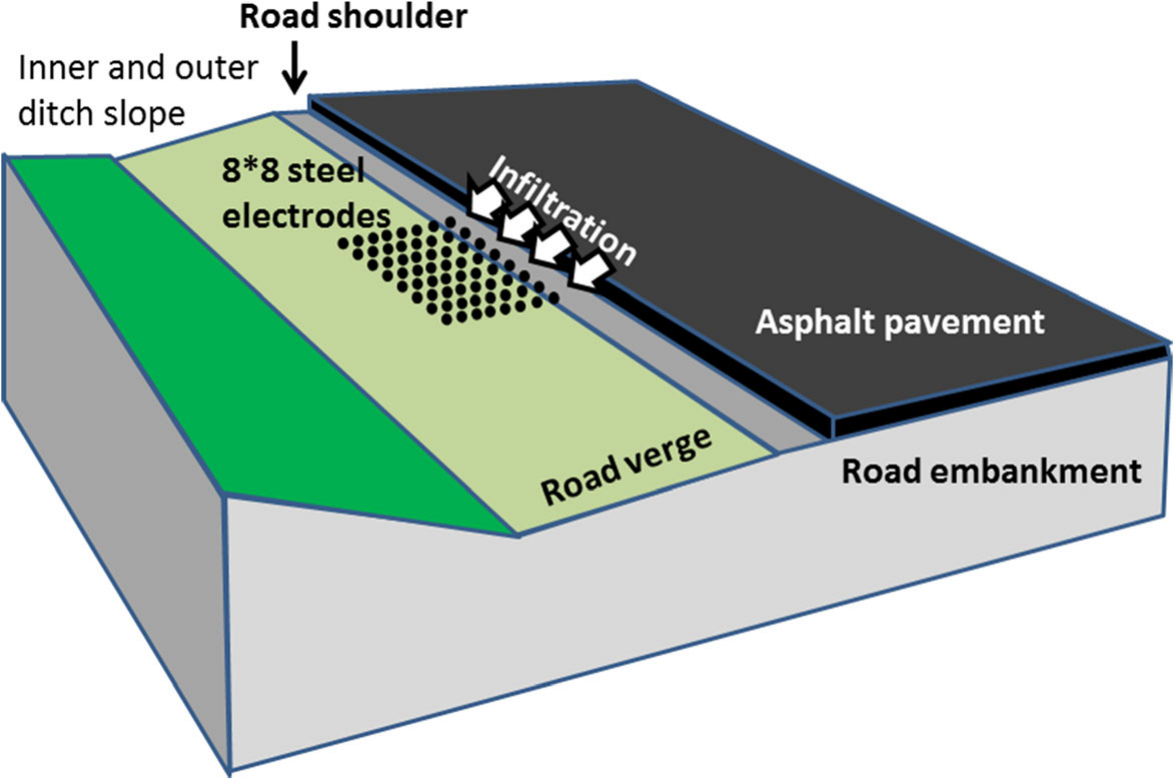
Road nomenclature and the geoelectrical array for tracer tests using pole-dipole measurements

In this study, a 64 electrode system (ABEM SAS4000) was used. Initially, various electrode arrays such as pole-pole, pole-dipole, dipole-dipole, and gradient, arranged in 2D and 3D electrode patterns comprising 64 electrodes, were tested at a site in Stockholm. According to Loke (1999), the pole and dipole configurations are more suitable for 3D measurements, since they can achieve better resolution along the borders of the measured volume. A flexible electrode and cable system was also built to enable testing of different electrode arrays. Finally, as a compromise between penetration depth, surface accuracy, and measurement speed, a symmetrical 8 × 8 electrode pattern using a pole-dipole array was selected, Fig. 1. A description of the pole-dipole electrode configuration is given in standard geophysical textbooks such as Reynolds (2011).

The apparent resistivity was calculated according to the following equation:

3 $$ \delta a=2\pi \times n\left(n+1\right)a\times \frac{V}{I} $$

where *a* is the distance between the potential electrodes and *n* is the spacing (*n* × *a*) between the potential and the closest current electrode. The outer fixed current electrode was positioned about 30 m from the test site.

Electrode spacings of 0.2 and 0.4 m were used. The penetration depth using 0.4 m electrode spacing was approximately 1.2 m, whereas the 0.2 m spacing only gave half this value.

Measurements were carried out before, during, and after the tracer infiltration tests. The time lapse from infiltration to the second measurement was usually between 30 and 120 min, and several different time lapse periods were tested. Each resistivity measurement took about 20 min.

Inverse finite difference modeling of resistivity data (245 blocks) was carried out in 3D using the software Res3DInv (Loke 2007). Modeled data were exported to ResCalc (©Olofsson), and Excel for further statistical analysis and finally presented in the software Voxler.

Transforming resistivity values into mass transport of water and pollutants is not possible since the initial conditions of humidity and porosity at each soil depth is not known. Heterogeneity of the road material will probably not give a Gaussian shaped plume. Therefore, pollutant spread is instead roughly based on a threshold change in modeled resistivity. The penetration depth with time represents the first change above the selected threshold value of modeled resistivity. The accuracy of the calculated resistivity decreases significantly with depth. Uncertainties is also related to the inverse modeling techniques used, and the fitting errors between measured and modeled resistivity data must be considered when a threshold value for resistivity changes is selected. Previous investigations (Lundmark and Olofsson 2007) have also shown that it is more difficult to identify resistivity changes in natural low resistivity environments. A threshold change of 25% is selected in this study considering the abovementioned uncertainties and previous experiments and calculations (Aaltonen and Olofsson 2002, Olofsson and Lundmark 2009).

### Tracer Tests

The simulation of an accident was carried out by instant release at the asphalt fringe of 50 L of sodium chloride solution with a chloride concentration of 1000 mg/L.

The infiltration capacity of the road shoulder was initially measured with a single-ring infiltrometer (Ø14.5 cm). Two infiltration studies were carried out at each location. Water was added, and infiltration was measured until a steady infiltration rate velocity was achieved. However, it was difficult to install the infiltrometer in the very coarse material near the shoulder of the asphalt fringe. Sampling of soils in the infiltration zone of the tracer test was carried out for texture and organic content analysis. Mixed soil samples were also taken from the infiltration zone at four distances from the road asphalt surface, for analyses soil water content before and after the simulated accidental spill of liquid. The soil water content was analyzed in the mixed soil samples by weighting before and after drying. Soil texture was analyzed by sieving and hydrometer analysis. Groundwater level measurements were carried out in existing groundwater tubes at four sites, which previously have had problems with water logging. There were no possibilities to make direct groundwater level measurements at all sites.

### Flow Modeling

#### One-Dimensional Modeling with CoupModel

General calculations of the transport time of water-borne pollutants in the unsaturated zone were carried out using simple analytical estimations and by dynamic modeling with CoupModel, a 1D physically based ecosystem model which can handle the interaction between soil, vegetation, snow, and atmosphere (Jansson and Moon 2001; Jansson and Karlberg 2004).

The analytical calculations of transport time in the ground were based on Darcy’s law assuming the soil is saturated, which gives faster flows than in unsaturated soils. The vertical flow time (*t*) between land surface and groundwater surface was calculated according to the following equation:

4 $$ t=\frac{d\times {n}_e}{K_s} $$

where *K*_*s*_ is saturated hydraulic conductivity (m/s), *d* is depth to groundwater surface, and *n*_*e*_ is effective porosity (m^3^/m^3^). The estimation of transport time by the Swedish Rescue Services integrated decision support unit (RIB) is generally based on this equation, using the assumption that the soil is homogeneous and saturated. This is a simplification and will give a worst case scenario based on a steady state flow. Analytical calculations were performed for clay, glacial till, sand, and gravel, using literature values of hydraulic conductivity and effective porosity.

**Fig. 2:**
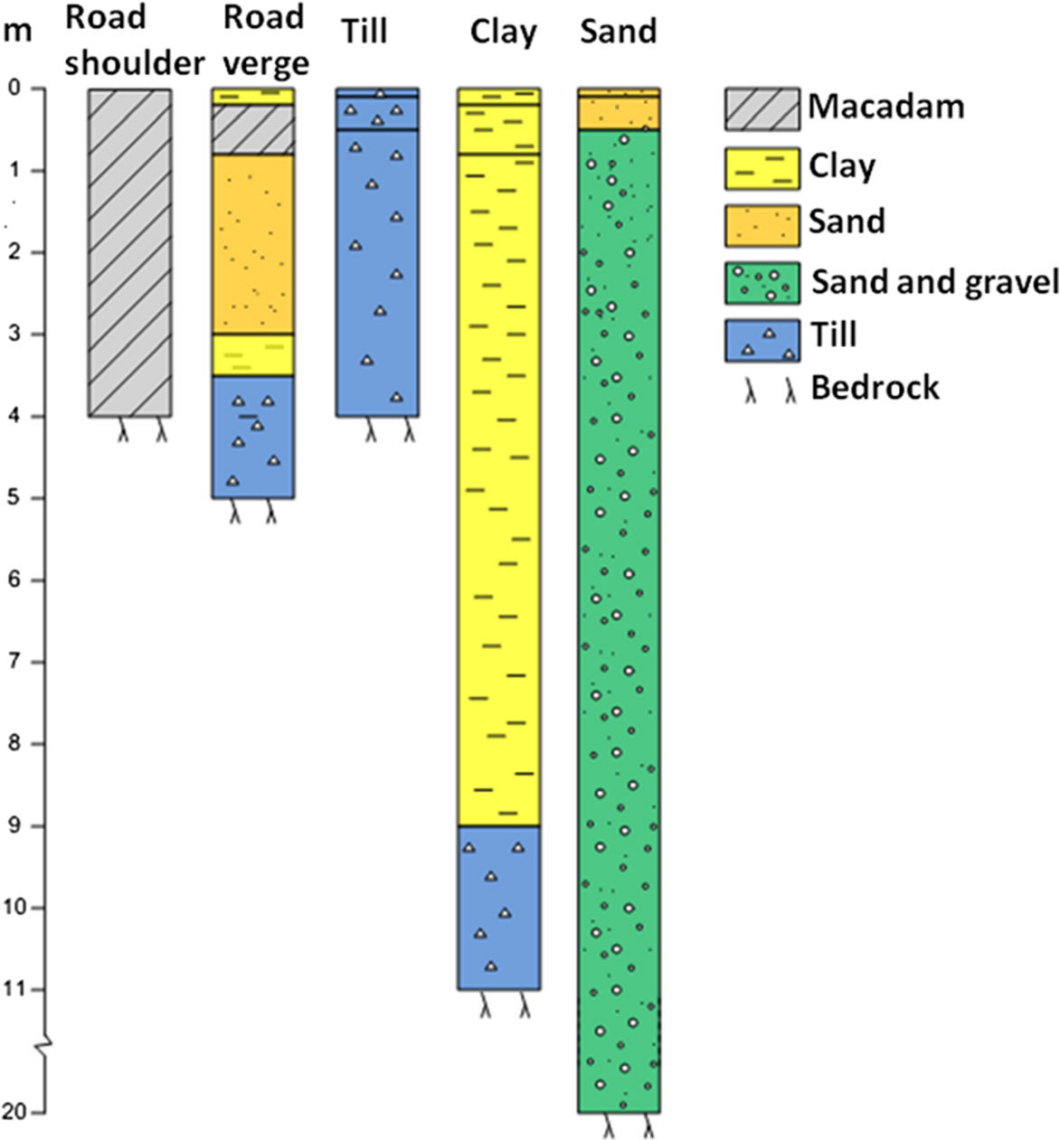
Stratigraphical soil profiles representing the five different soils included in dynamic modeling with CoupModel

Dynamic modeling of the movement of water and chloride in different soil profiles using CoupModel is based on the Richards’ equation for unsaturated flow (Richards 1931) and the Brooks and Corey (1964) water retention function. Generic soil descriptions of soil types that can be found along roads in central and southern Sweden were used for the modeling. The generic soil descriptions included a possible vegetated stratigraphy, hence taking possible vertical heterogeneity into consideration. Three natural generic soil environments were used for the 1D modeling (clay, glacial till, sand) and two constructed soil environments (roadside verge and asphalt fringe. The soil stratigraphies are shown in Fig. 2.

Data on the hydraulic properties of clay, till, sand, and the verge were taken from the Swedish soil database, which is integrated in CoupModel as described by Lundmark and Jansson (2008). Data on the hydraulic properties of the asphalt fringe were taken from Ekblad and Isacsson (2007), representing typical bare crushed rock (Table 1). All soil profiles were assumed to be covered with grass, except for the asphalt fringe that had no vegetation cover. Groundwater level was assumed to be 2 m below the land surface for all soils.

**Table 1:** Soil hydraulic properties used for the CoupModel-simulations, represented as estimated coefficients for the Brooks and Corey water retention function based on Lundmark and Jansson (2008) and Ekblad and Isacsson (2007)

	Depth (m)	Pore size distribution index (*−*)	Air entry (cm)	Saturation (vol%)	Residual water (vol%)	Wilting point (vol%)	Hydraulic conductivity (mm/day)
Road shoulder	–	0.75	1.9	23	1	2.45	864,000
Macadam in road verge	–	2	4	40	1	1	1000
Clay	0–0.2	0.06	2.9	52	1.5	15	833
	0.2–0.8	0.05	2.6	50	2.6	19	511
	>0.8	0.04	3.2	51	0.7	20	318
Sand	0–0.1	0.54	18	49	7.6	3.5	2469
	0.1–0.5	0.71	20	44	7	2.9	2487
	>0.5	0.97	19	42	4.6	2.3	2641
Sand and gravel	–	1.1	19	35	2.3	0.01	15,000
Till	0–0.1	0.27	4.9	72	6	4.9	2864
	0.1–0.5	0.26	10	48	2.6	5	695
	>0.5	0.19	10	36	0.7	4.5	142
Bedrock	–	0.01	5	5	0.01	2	0.01

Climate data from central Sweden are used as driving variables in CoupModel. A 4-year period was simulated (1 Sept. 2002–30 Aug. 2006) using time steps of 1 h. A simulated infiltration of 50 L saltwater (chloride concentration 1000 mg/L) on an area of 0.216 m^2^ was modeled. The front of the chloride plume (concentration <2 mg Cl^−^/L, which is similar to an unaffected soil) was studied at different time periods and compared with the analytical calculations based on the resistivity measurements and inverse modeling.

#### Two-Dimensional Flow Modeling

COMSOL Multiphysics 5.2, a commercial finite element multi-physics modeling software, was used to model 2D water flow next to two different types of roads, one old consisting mainly of natural geological material found in the road surroundings and one modern built up of coarse and crushed material according to Swedish Transportation Administration standards. The Richards’ equation was used for the unsaturated flow, coupled to the Heat-Transfer-in-Porous media function from the software’s physics library (COMSOL 2016). No flow boundary defined the asphalt layer and sides of the road section. The groundwater level was set at 2 m below the surface and flow was modeled only within the unsaturated part. Both temperature and precipitation were set as temporal piece-wise functions for the upper boundary. Permeability of the soils was estimated from sieve analysis data and the Hazen (1892) equation:

5 $$ K=C{\left({D}_{10}\right)}^2 $$

where *D*_10_ is sieve size (mm) corresponding to the 10% of retained material and *C* is Hazen coefficient (range 0.4–1.2). The lowest coefficient value is used in calculating the minimum permeability and the highest in calculating the maximum permeability.

**Fig. 3:**
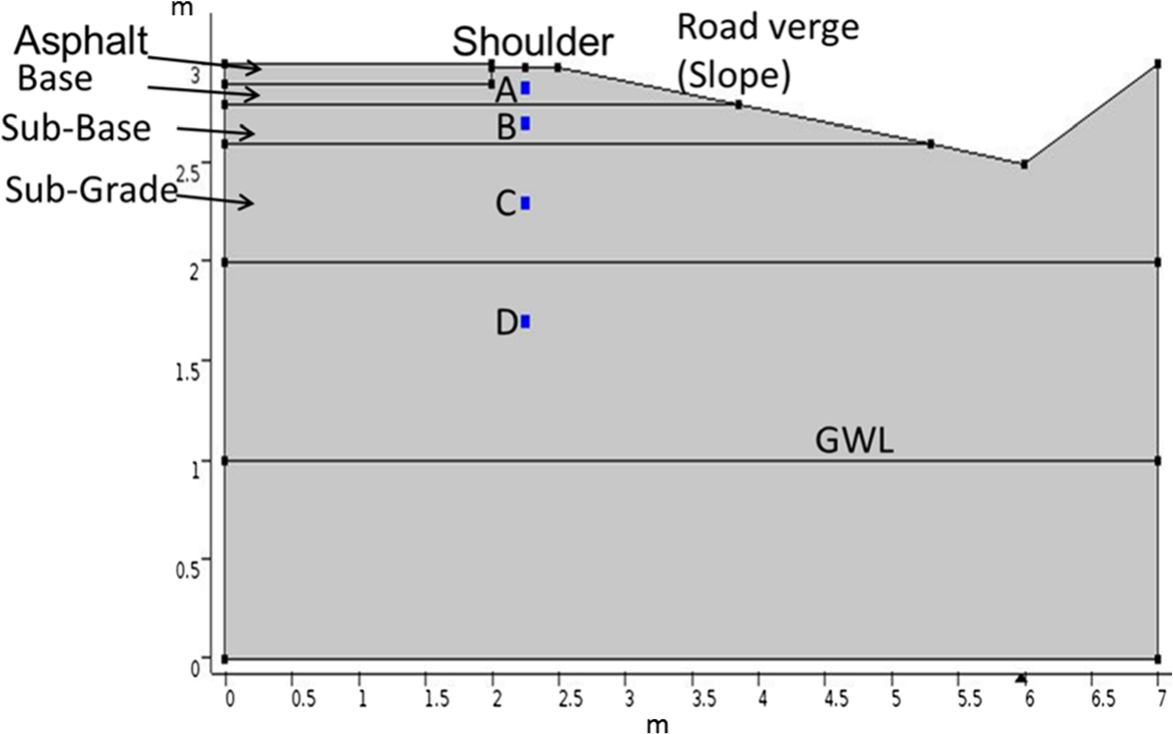
Geometry of part of a typical modern road section used in COMSOL modeling

Soil water characteristic parameters for the retention model were estimated using a pedotransfer function as described in Benson et al. (2014) and from the particle size distribution and uniformity coefficient. The construction of the studied roads was unknown, especially for older roads. Therefore, a generalized road section typical for Swedish roads, down to a depth of 3 m, was formulated for the 2D modeling, shown in Fig. 3.

Based on all soil samples taken from the different road shoulders, the permeability ranged between 0.4 × 10^−14^ and 1.03 × 10^−9^ m^2^. Other parameters were based on the literature; for road layers, the parameters were taken from Hansson et al. (2005) and for soil layers from Lundmark and Jansson (2008). See Table 2 for the list of parameters used in the COMSOL simulation.

**Table 2:** Hydraulic properties of soil and parameters used in 2D modeling, based on Lundmark and Jansson (2008), Hansson et al. (2005), and soil grain size distribution

	Parameter	Embankment (natural soil)	Sub-grade	Sub-base	Base	Asphalt
Modern road	Saturation *θ*_s_ (%vol)	40	38	23	21	5
	Residual water *θ*_r_ (%vol)	6	4	3	2	1
	Permeability *K* (m^2^)	0.4 × 10^−14^	1.03 × 10^−9^	1.03 × 10^−9^	1.03 × 10^−9^	1 × 10^−17^
	Pore size distribution *λ*	0.37	0.42	0.42	0.67	3
	Air entry *ψ*_a_ (cm)	0.006	0.002	0.002	0.002	1
	Specific gravity (kg/m^3^)	1200	1200	1600	1800	2300
	Saturation *θ*_s_ (%vol)	40	38	30	30	5
Old road	Residual water *θ*_r_ (%vol)	6	4	2	2	1
	Permeability *K* (m^2^)	0.4 × 10^−14^	1.7 × 10^−11^	1.7 × 10^−11^	1.7 × 10^−11^	1 × 10^−17^
	Pore size distribution *λ*	0.37	0.42	0.42	0.67	3
	Air entry *ψ*_a_ (cm)	0.006	0.002	0.002	0.002	1
	Specific gravity (kg/m^3^)	1200	1200	1600	1800	2300

The flux used was 1 kg/(m^2^·s), and flux was only applied at the shoulder part for a limited time (80 and 120 min), in order to simulate an accident lasting 100 and 500 min for modern and old road, respectively.

### Field Areas

**Fig. 4:**
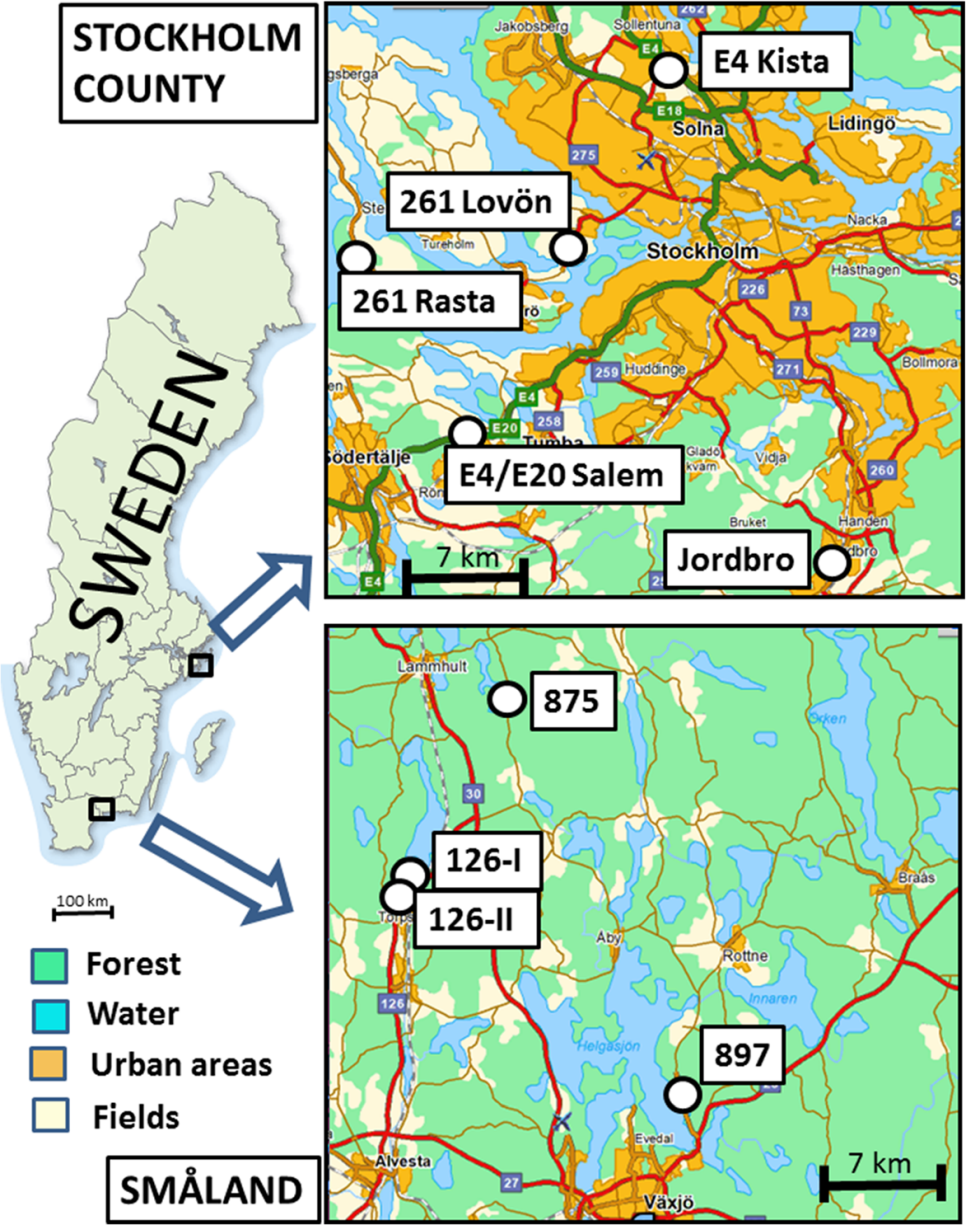
Field sites in southern Sweden (Småland) and in Stockholm County

**Fig. 5:**
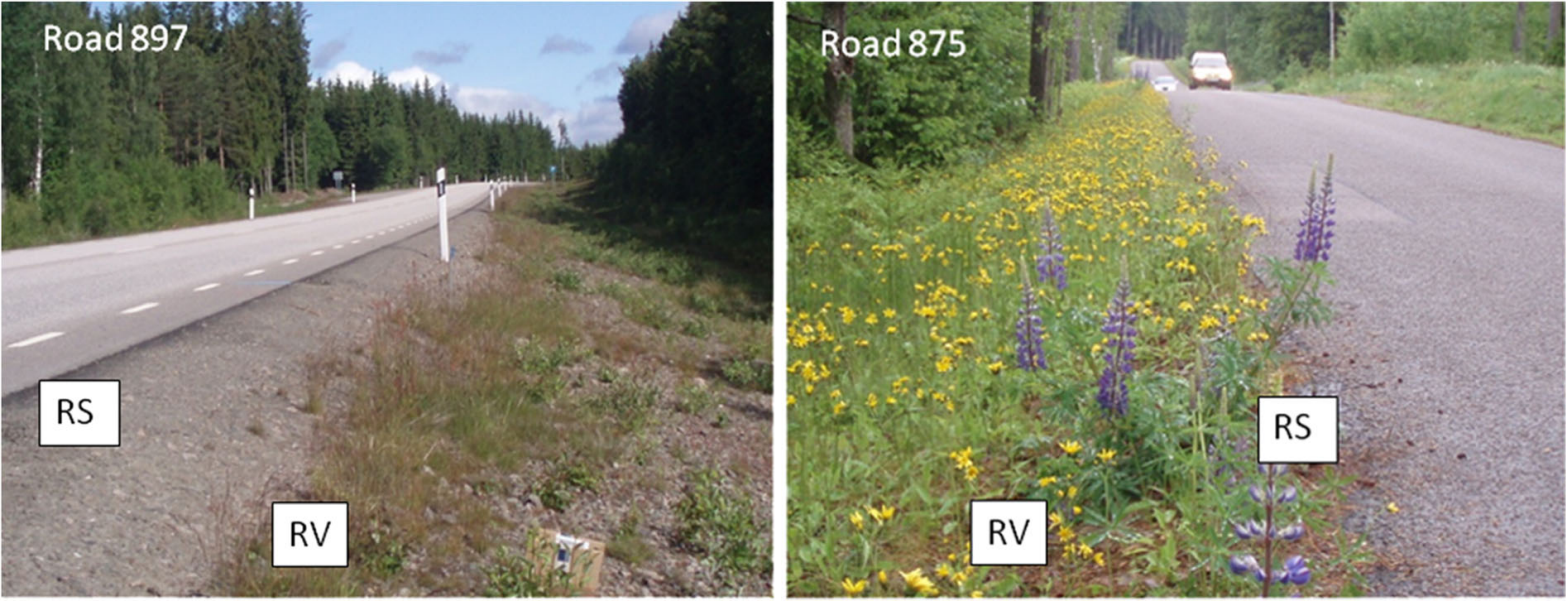
Two typical roads in southern Sweden, a recently constructed macadam (Road 897; *left*) and an old road mainly consisting of natural mixed material (Road 875; *right*). (*RS* = road shoulder, *RV* = road verge)

Field measurements were carried out along old and modern roads in southern Sweden (at four locations in Småland, north of Växjö) and central Sweden (five roads around Stockholm), Fig. 4. The roads represented different categories, from minor roads built up of local soils found in the surroundings to modern roads built up of macadam, which usually consists of crushed and angular coarse aggregates, such as gravel and stones, Fig. 5. The roads were classified according to their stratigraphy, traffic, width, local geology, and hydrology, Table. 3. The natural geology at Småland commonly consists of till, whereas the sites around Stockholm comprise areas with till, clay, sand, and gravel. The field measurements were made during summer (in June and August), representing natural dry conditions, and the top layer of the infiltration area was assumed to be unsaturated at the time of the tracer tests.

**Table 3:** Field site characteristics. *ADT* average daily traffic, *Macadam* crushed and angular coarse material (gravel and stones)

Measurement site	Coordinates (RT90)	Inner ditch slope (%)	Material in the inner ditch slope	ADT Cars/trucks	Road width (m)
Road 897, Skirsvad	6313443/1441421	16	Macadam	2940/190	9
Road 875, Berghem	6335960/1431179	10	Sandy till	120/6	5.5
Road 126 I, Torpsbruk	6325274/1424766	30	Macadam	1040/200	8
Road 126 II, Torpsbruk	6325000/1424690	24	Macadam	1040/200	8
Road 261 Lovön	6578978/1617114	19	Clayey silty sand	16,690/1150	11.2
Road 261 Ekerö Rasta	6578253/1604370	14	Gravelly sand	2630/200	7
G:a Nynäsvägen, Jordbro	6558840/1632336	15	Gravelly sand	8140/690	7
Road E4/E20 Salem	6567268/1611162	28	Macadam	24,440/2370	11.5
Road E4 Kista	6589500/1622210	20	Macadam	36,500/2850	15

The resistivity measurements were carried out using both the 0.2 and 0.4 m electrode spacing.

## Results and Discussion

### Infiltration

**Fig. 6:**
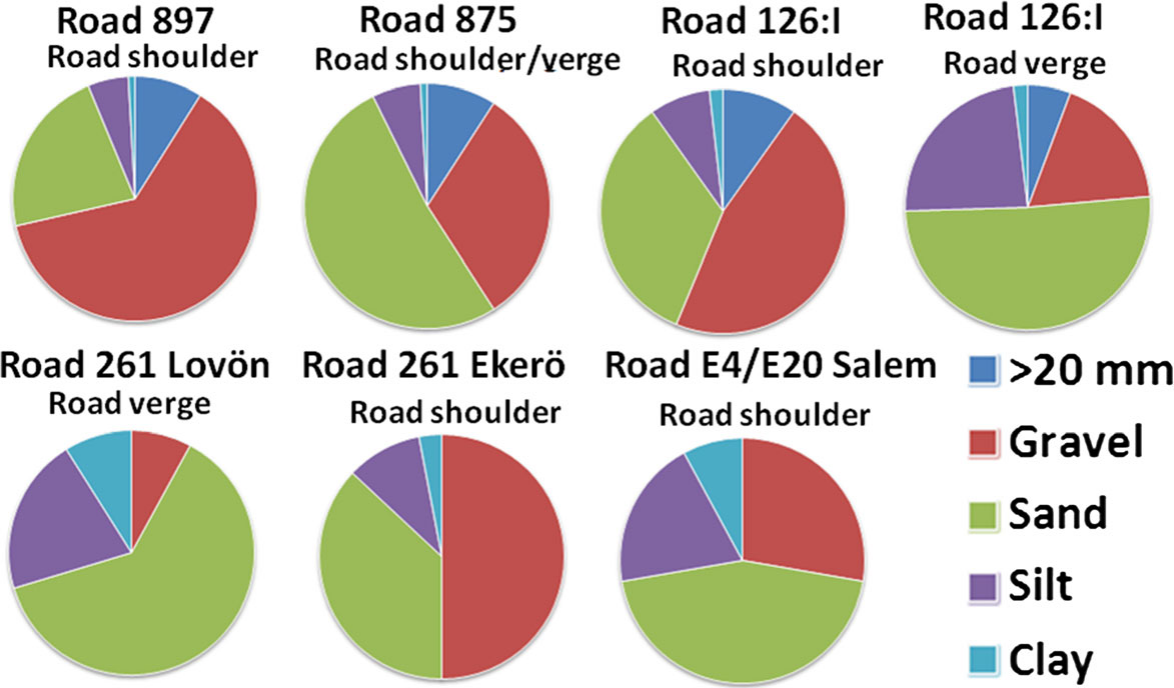
Grain size distribution from sieving and hydrometer analysis of soil samples from road shoulders and verges in Småland and Stockholm County

Results from the measurements of infiltration and texture analyses of soils are shown in Table 4 and Fig. 6. There were often considerable variations in infiltration capacity between the two infiltration tests carried out at each site. This is probably an effect of strongly heterogeneous conditions, but can partly also be an effect of measurement problems with leaking contacts between soil and infiltrometer due to the coarse soil material. However, these problems were similar at all sites, which may have given a minor systematic error but did not significantly impact the overall results, which were based on a comparison between the sites. The infiltration capacity of the road shoulder was usually within the range 10^−5^ to 10^−6^ m/s. Calculations of the relative infiltration capacity of all test sites indicated that it varied by a maximum of 52-fold between the highest and lowest. The variation of the infiltration capacity at each road is usually small (one–twofold) compared to the variations between the roads. The lowest infiltration capacity was found next to the old Road 261 in Stockholm County, while the highest capacity was found next to a recently constructed modern, coarse-textured Road 897 in southern Sweden, Table 3. The difference in infiltration capacity between these roads was about 40- to 50-fold. The main explanation is the more fine-grained material along the older road in Stockholm County. Correlation analysis revealed that the infiltration capacity was inversely correlated to the content of fine-grained particles, mainly silt and clay. The results of some of the grain size analyses are presented in Fig. 6.

**Table 4:** Results from infiltration and texture analyses of soils (*gr* gravel, *sa* sand, *si* silt, *cl* clay, *ti* till)

Measurement site	Infiltration capacity at road shoulder (m/s)	Factor difference in infiltration capacity to lowest capacity (−)	Texture road shoulder/inner ditch slope	Organic content (% of dry weight) road shoulder/inner ditch slope	Groundwater level (cm below surface)
Road 897, Skirsvad	3.3 × 10^−5^	40	sa gr	1.5	60
	4.3 × 10^−5^	52		2	
Road 875, Berghem	1.5 × 10^−5^	18	gr sa ti	1	30
	2.3 × 10^−5^	28		4.7	
Road 126 I, Torpsbruk	2.0 × 10^−5^	24	sa gr	3.1	240
			si sa gr	0.4	
Road 126 II, Torpsbruk	8.3 × 10^−7^	1	sa gr	3.1	40
	1.5 × 10^−5^	18		4.4	
Road 261, Lovön	8.3 × 10^−7^	1	si sa	4	
	1.6 × 10^−6^	2	cl sa ti	6.5	
Road 261, Ekerö Rasta	1.0 × 10^−5^	12	sa gr	2.4	
	2.0 × 10^−5^	24	gr sa	3.8	
G:a Nynäsvägen, Jordbro	8.3 × 10^−6^	10	gr sa	3.7	
	5.0 × 10^−6^	6	si sa	4.2	
Road E4/E20 Salem	5.0 × 10^−6^	6	cl gr sa	6.6	
	2.0 × 10^−5^	24	cl si sa	4.4	
Road E4 Kista	6.7 × 10^−6^	8	si sa	5.6	
	1.5 × 10^−5^	18	cl gr sa	1.3

Analyses of the organic content of the soils were carried out since organic matter may prevent some compounds such as heavy metals to reach groundwater. The organic matter content in the shoulder at the asphalt fringe was fairly low, at most 6%, along the major highway E4, both north (Kista) and south (Salem) of Stockholm. Road 261 at Lovön, which passes through an agricultural area, had similar organic matter content. There was a clear inverse correlation between content of gravel and organic material in the soil and a positive correlation between organic matter and the content of sand, silt, and clay. The water content in the road shoulder varied between 5 and 12%. The overall natural moisture of the road verge and the variation in water content (2–35%) were considerably higher than in the road shoulder. The highest water content (35%) was found along Road 126 in Småland, at distances of 70 and 150 cm from the pavement. Road 897 in Småland was rather dry, due to high infiltration in the coarse material, which makes this site highly vulnerable to pollutant spread to groundwater.

### Resistivity Measurements

The coarse macadam material in the road led to rapid infiltration of conductive tracers. Therefore, use of the 0.2 m electrode spacing cables gave too limited depth penetration, and the following geophysical results relate to measurements made with 0.4 m electrode spacing.

**Fig. 7:**
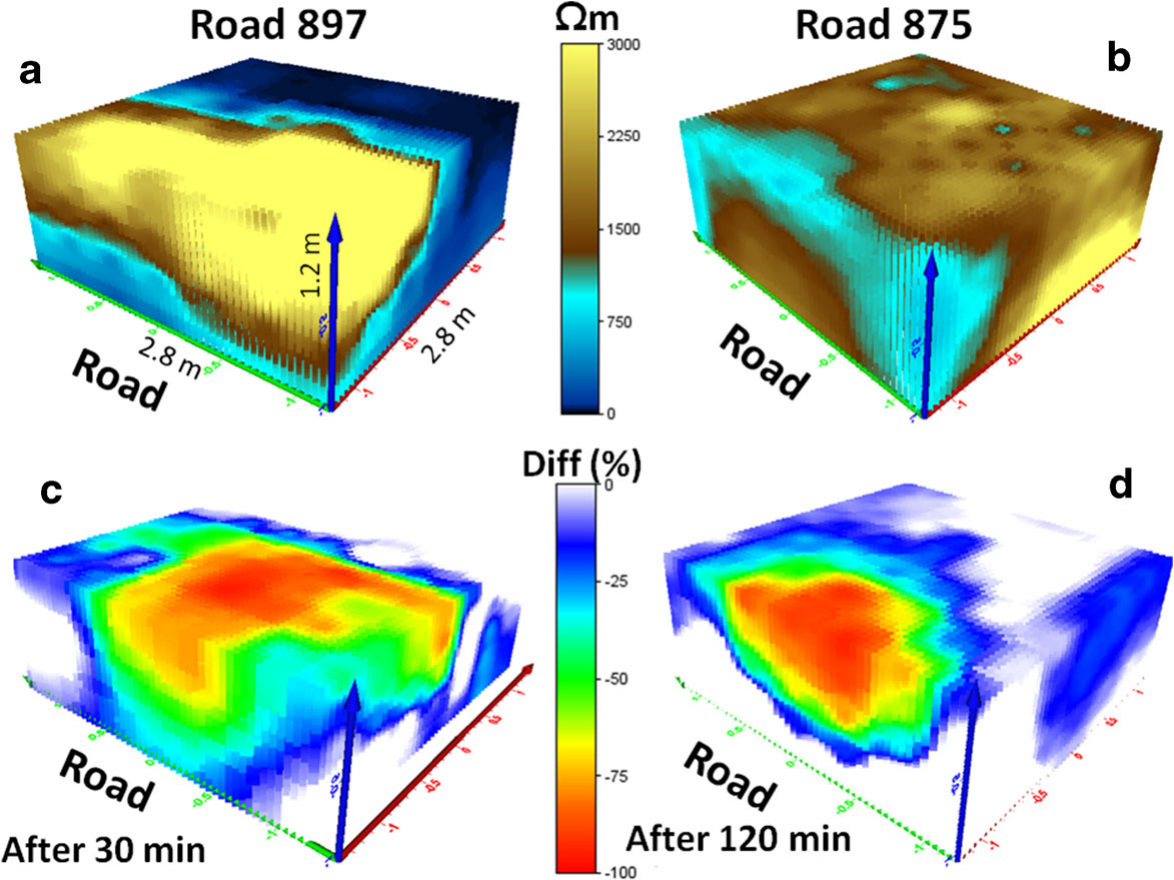
Results of three-dimensional resistivity modeling next to two typical roads in southern Sweden: a modern road built up of macadam (Road 897) and an old road constructed from local soils (Road 875). **a**, **b** represent initial resistivity conditions; **c**, **d** represent time-lapse calculations showing percentage change in resistivity 30–120 min after the tracer tests

The results are presented as modeled 3D plots showing the variation in resistivity before and after the simulated spill of liquids (tracer tests). The time-lapse differences in resistivity varied from 30 min to 2 h, depending on the variation in infiltration capacity. Major results for two typical roads, in Småland (Road 897) and Stockholm (Road 875), are presented in Fig. 7. These roads represent two different categories of construction, a modern road (Road 897) with an embankment of macadam which stretches out on the inner ditch slope, and a small older road (Road 875) with unknown embankment, probably similar material as in the surrounding geological formations. The resistivity measurements which were carried out before infiltration probably represent the road embankment material with natural moisture content (0–35%). Road 897 has high resistivity close to the asphalt fringe, probably due to the embankment of macadam. Downslope resistivity decreased significantly due to high content of clay or water. A shallow groundwater level (about 60 cm beneath the road shoulder) was confirmed by measurements in groundwater wells. The resistivity close to Road 875 indicated a shallow zone of resistivity 500–1000 Ωm, due to the sandy road shoulder.

The calculated changes in resistivity after discharge of saltwater next to the modern Road 897 showed that water infiltrated and percolated downwards in the road shoulder, mainly within 2 m from the asphalt fringe, with a maximum redistribution of salt at about 0.5 m from the asphalt fringe, Fig. 7. Almost no changes were noted at longer distances from the asphalt, where the natural resistivity was low. Changes due to conductive tracers are generally difficult to identify in low resistivity material (Lundmark and Olofsson 2007).

Road 875, which is built up of natural material, originally had lower resistivity close to the asphalt fringe. The infiltration zone was almost totally located within 1 m from the asphalt fringe, Fig. 7. The gentle inner ditch slope (10%) and dense grass vegetation permitted slow infiltration without lateral downslope spread. The infiltration and percolation in the natural material along Road 875 was significantly slower than in the macadam in Road 897.

**Fig. 8:**
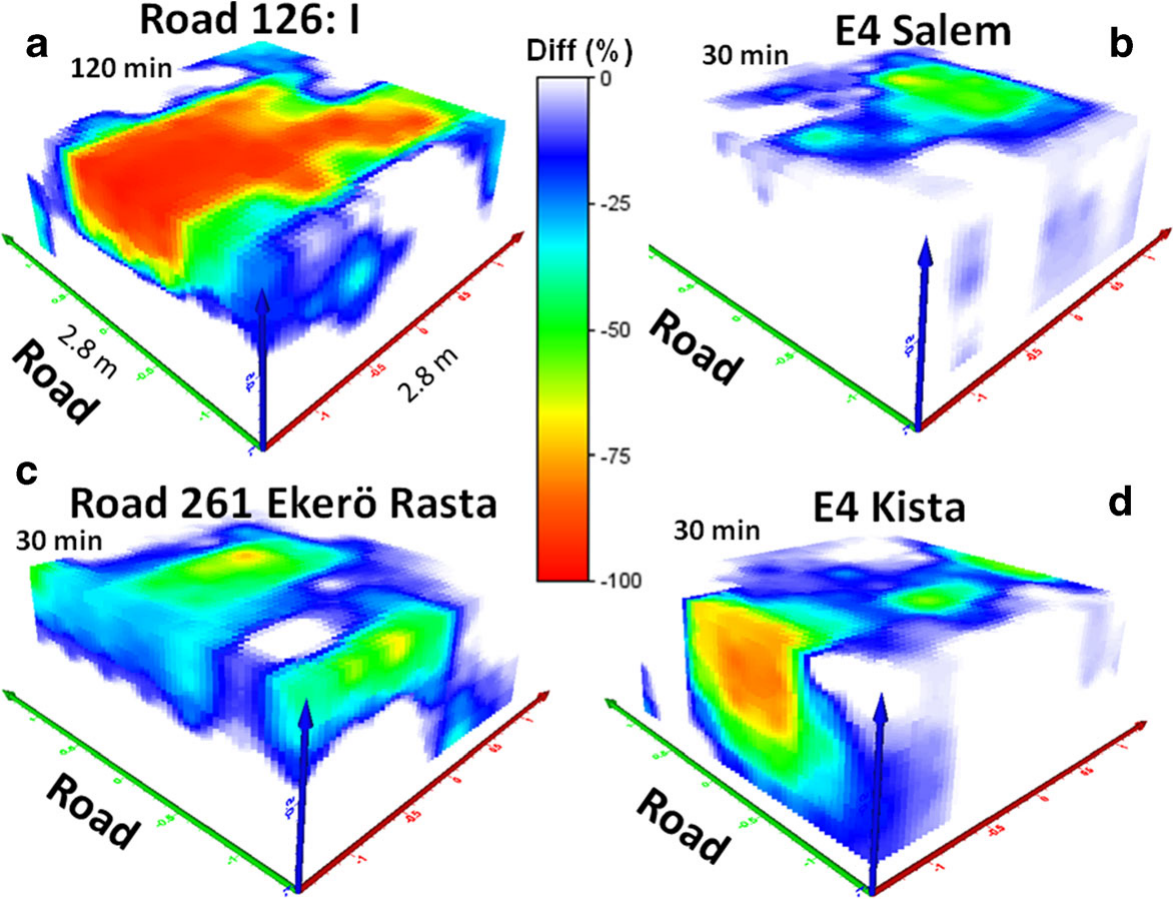
Results of three-dimensional resistivity time-lapse modeling at four typical roads in southern and central Sweden, showing percentage change in resistivity 30–120 min after tracer tests

At several study sites in the Stockholm area, some overland flow was noted during the tracer tests and some water infiltrated downslope from the electrode system, e.g., at G:a Nynäsvägen. This was also shown by the resistivity analyses. G:a Nynäsvägen is located on a natural sandy glaciofluvial deposit (esker), and the inner ditch slope was moderate (15%), and hence overland flow was not expected here. It is clear that high inner ditch slopes sometimes favor overland flow and decrease infiltration in the road verge. This was observed at E4 Salem, with an inner ditch slope of 28%, where most of the tracers probably infiltrated downslope from the electrode system, since only small resistivity changes were identified more than 2 m from the asphalt fringe. At Road 126-I in Småland, the steep inner slope (30%) led to lateral downslope spread of the tracer infiltration, Fig. 8. However, no clear correlation was found between surface runoff and soil texture, infiltration capacity, or water content. The measurement site at E4 Kista is also located on a sandy deposit, but the embankment is made of macadam. No surface runoff was noted here and most water infiltrated close to the asphalt fringe, Fig. 8. At Road 261, Ekerö Rasta, it was clear that tracer partly ran along the road, probably on the asphalt pavement, before it entered the road shoulder and road verge, as the infiltration occurred in two sub-areas, Fig. 8.

**Fig. 9:**
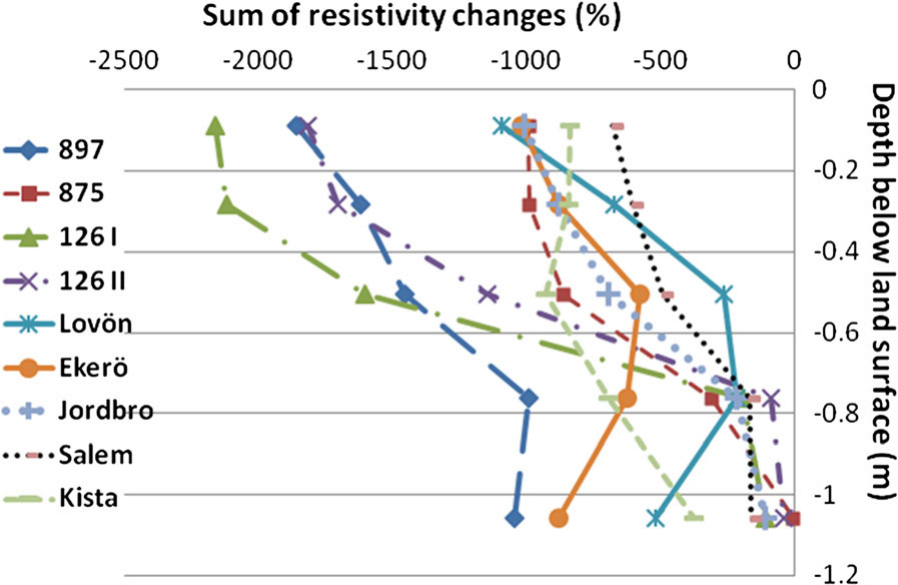
Total sum of changes in resistivity (%) during time-lapse measurements as a function of depth below the ground surface (m) at the different road sites studied in southern and central Sweden

The resistivity distribution with depth for the different sites is shown in Fig. 9. All measurement sites showed a clear decrease in resistivity due to infiltration down to a depth of 1.2 m, which was the maximum measurement depth for this electrode configuration. Road 897, which had the coarsest material in the road shoulder and a road embankment of macadam, also showed the largest decrease in resistivity with depth. Despite coarse material in the surface, Road 261 (Ekerö Rasta) showed a small change with depth, since the old road is built up of natural material. The significant decrease of resistivity in Road 126 (I and II) in Småland, which mainly occurred at shallow depth (<0.6 m), was probably due to the presence of drainage pipes on each side of the experimental area, which drained the infiltrated tracer horizontally, and hence the functioning of the drains was evident.

Correlation analysis was carried out between the mean decrease in resistivity and the fraction of various grain sizes (Spearman’s rho, significance level *p* = 0.05). A strong negative correlation was found between the decrease in resistivity and the more fine-grained soils, i.e., clay, silt, and sand (−0.81, −0.83, and −0.68, respectively), but a positive correlation between decrease in resistivity and the amount of coarse-grained soils present, i.e., gravel (+0.82) and stone (+0.68). The largest decrease also occurred at shallow depths and the change decreased with depth. The difference between the resistivity measurements carried out soon after infiltration compared with after some hours was very small.

### Modeling of Transport Time

#### Transport Time Based on Resistivity Measurements

A rough estimation of transport time was made based on resistivity measurements. Due to uncertainty within the resistivity measurements, modeling technique and low accuracy with depth, described in the methodology chapter, and considering the fitting error of the resistivity modeling, a threshold value for the pollutant plume was set to a decrease in resistivity of more than 25%. This was in line with previous calculations and experiences in Sweden (Aaltonen and Olofsson 2002, Olofsson and Lundmark 2009). The first time the decrease of resistivity with depth exceeds the threshold value therefore roughly represents the actual percolation at that moment. The infiltration at some of the sites was so rapid that the actual penetration depth could not be measured with the measurement array used. Table 5 presents the estimated penetration depth, percolation velocity, and percentage of affected soil at 1.2 m depth. The resistivity measurements indicated rapid downward, gravity-driven flow, amounting to 1.4–8.6 × 10^−4^ m/s. Road 897 in Småland and Road 261 at Ekerö Rasta exhibited the fastest infiltration rate, with 1 m depth reached within 20 min. More than 30% of the soil volume at 1.2 m was affected by the tracer at these sites, which was calculated from the deepest grid block elements.

**Table 5:** Calculated flow velocity, transport time, and volume of soil affected at the different measurement sites. Depth penetration is the depth where resistivity decreased by 25% in at least one grid element of the resistivity. For very permeable soils with rapid percolation, the penetration depth (>1.2 m) was calculated based on the depth penetration between two measurements. Time is the time lapse between infiltration and the second measurements. Volume of soil affected is the relative amount (%) of the model block affected at depth 1.2 m, i.e., where the resistivity had decreased by more than 25%

Measurement site	Depth penetration (m)	Time (h)	Percolation velocity 10^−4^ (m/s)	Transport time to depth 1 m (min)	Amount of soil affected at 1.2 m depth (%)
Road 897, Skirsvad	1.57	0.5	8.6	19	35
Road 875, Berghem	0.92	2	1.4	130	0
Road 126 I, Torpsbruk	1.39	2	1.9	86	4
Road 126 II, Torpsbruk	0.84	1	2.2	71	0
Road 261 Lovön	1.52	0.5	8.3	20	18
Road 261 Ekerö Rasta	1.55	0.5	8.6	19	31
G:a Nynäsvägen, Jordbro	0.91	0.5	5.0	33	0
Road E4/E20 Salem	0.70	0.5	3.9	43	0
Road E4 Kista	1.38	0.5	7.8	22	10

#### Transport Time Based on Analytical Calculations and Modeling

**Fig. 10:**
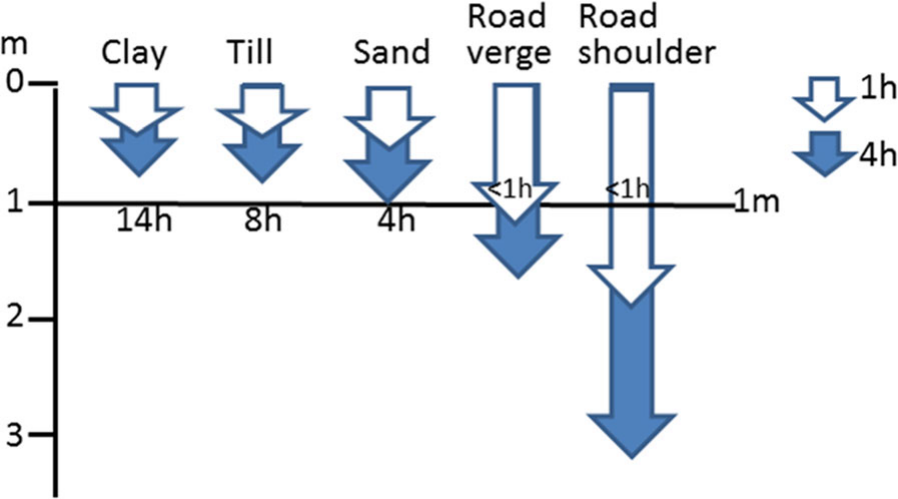
Calculated depth of the chloride front beneath the land surface in different materials at 1 and 4 h after a simulated discharge using 1D CoupModel. The initial chloride concentration was similar to that in field tracer tests (1000 mg/L). The calculated transport time to a depth of 1 m is also presented

The resistivity measurements were compared with the results of analytical calculations and results from 1D and 2D dynamic modelings using parameters from type soils based on typical Swedish soil stratigraphies and typical modern road sections (Lundmark and Jansson 2008, Hansson et al. 2005). The analytical calculations are a worst case scenario and represent the first arrival time, assuming that the soils are saturated, while the dynamic 1D modeling also considers variation in water content with time, different hydraulic properties, evapotranspiration, and uptake in vegetation. In fine-grained soils (clay and till), the analytical calculations gave a considerably longer transport time down to a depth of 1 m (1 year) than the dynamic model (14 h), whereas for sand and gravel, analytical calculations gave a much shorter transport time (max. 0.5 h) compared with the dynamic 1D modeling (4 h). The analytical calculations using fully saturated soil conditions therefore gave unrealistic travel times compared with measured resistivity values, but also in relation to previous studies of infiltration in natural soils in Sweden (e.g., Maxe and Johansson 1998). The dynamic 1D modeling, which was also applied to the constructed parts of the road, showed that the tracers could reach to more than 3 m depth within 4 h due to infiltration in the road shoulder**,** Fig. 10.

**Fig. 11:**
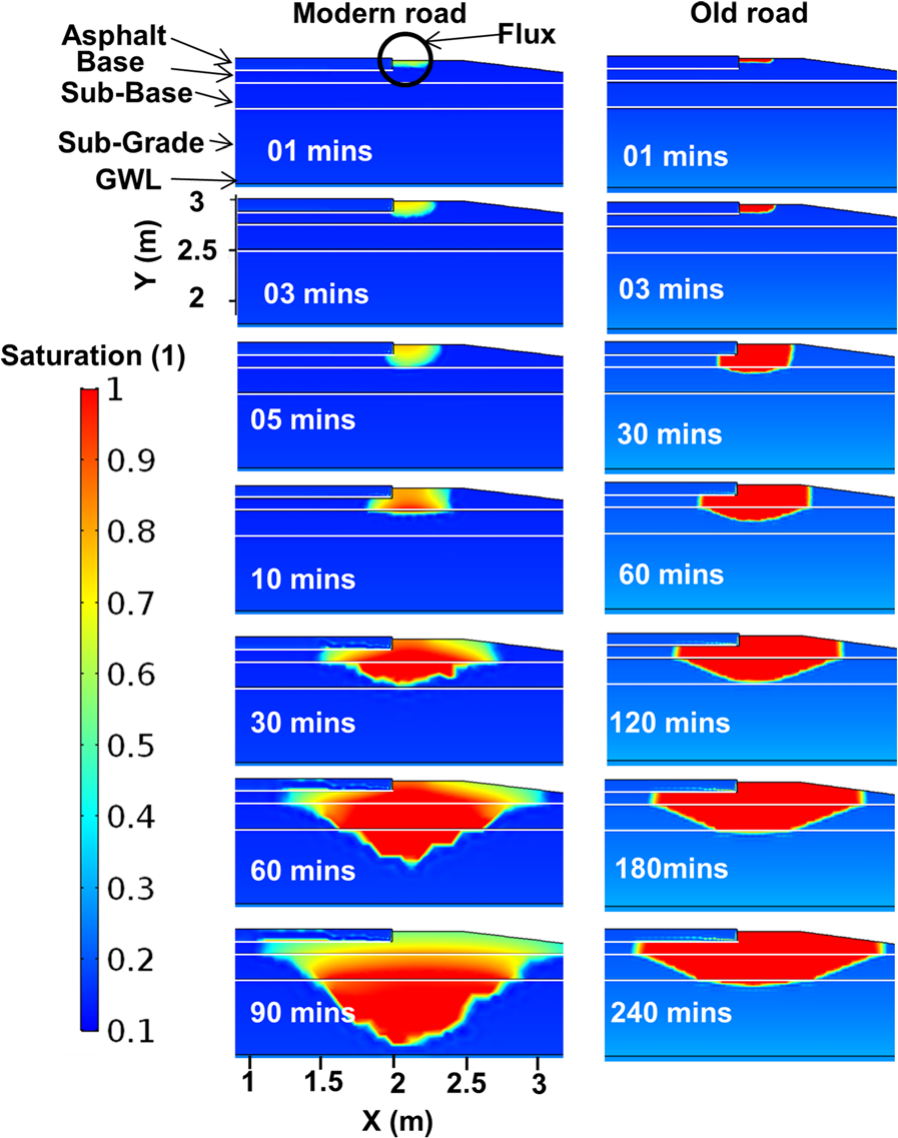
Two-dimensional simulation results for two road types in COMSOL Multiphysics 5.2, a modern road built up of rather coarse material (*left*) and an old road (*right*) consisting of local natural materials, showing the saturation changes due to added flux at the shoulder

Therefore, on modern roads made from coarse rock fragments, the time window for action after traffic accidents, such as digging, is in fact very limited. Similar results were found in the 2D COMSOL modeling. The change in saturation for two different types of roads, simulated for two different periods, is shown in Fig. 11.

**Fig. 12:**
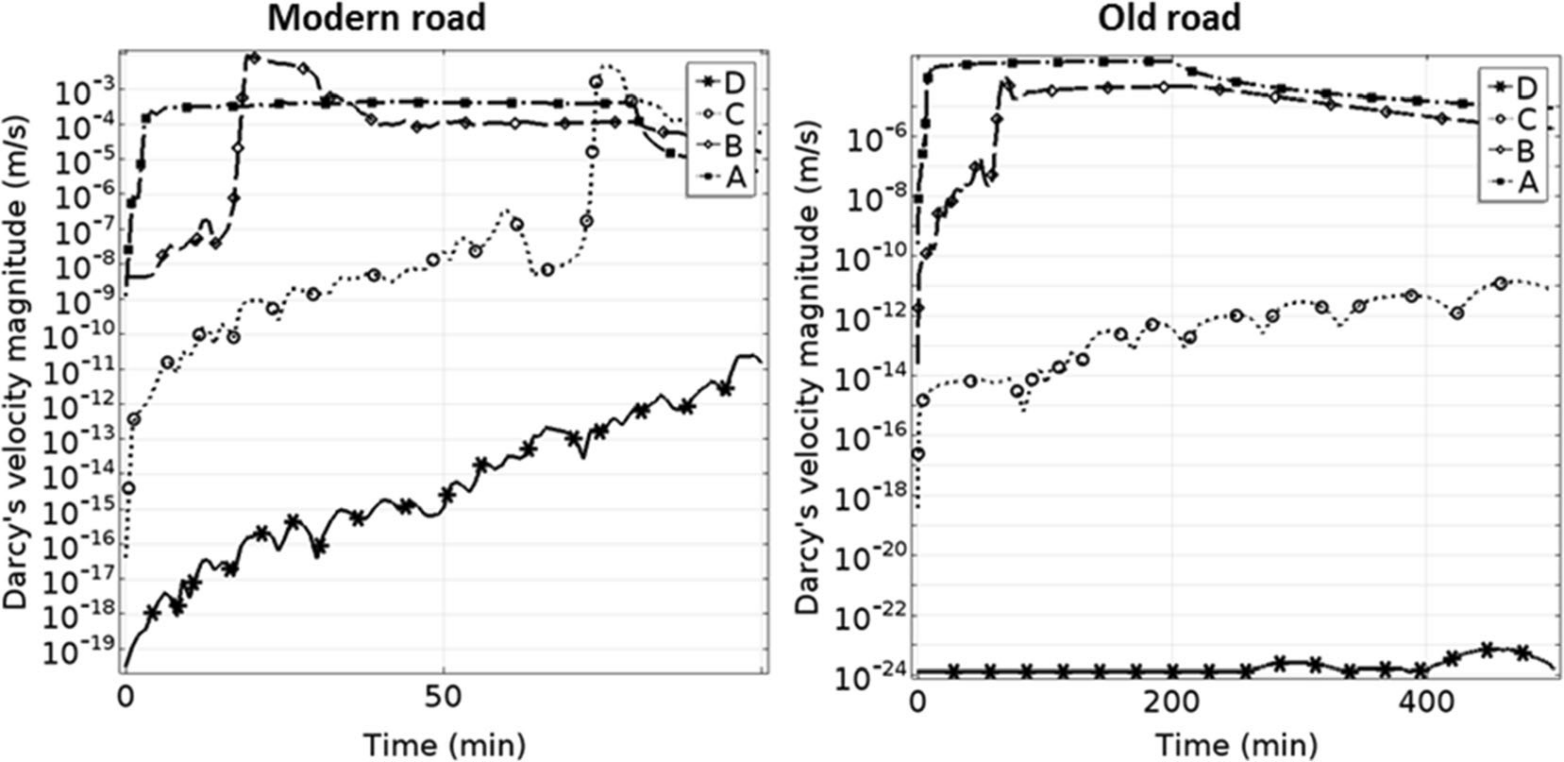
Flow velocity variations over time at different points marked *A*, *B*, *C*, and *D* in Fig. 3, for two types of road, a modern road consisting of coarse crushed material (*left*) and an old traditional road constructed from local soils (*right*)

The modern road (similar to Road 867) was modeled for 100 min and the old road (similar to Road 875) was modeled for 500 min. The saturation change in the modern road percolated rapidly to deeper layers until it reached the sub-grade material, where the permeability was lower. In the old type road, where the road material is mostly taken from surrounding soils, the spread of the infiltrating water was larger and the percolation was much slower. The infiltrating water had a tendency to flow horizontally under the asphalt layer and within the sub-base material. In the uppermost 20 cm of the road shoulder at the modern road, the simulation indicated faster flow movement downward compared with the old road, but with less saturation due to presence of more granular material. Within 5 min, the flow reached the sub-base material, while in the old road type, it took 30 min for the flow to reach this depth. In the modern road, the simulated flow reached the sub-grade material in less than 30 min, whereas in the old road, it took around 2 h. Within less than 60 min, the flow in the modern road reached a depth of 1 m, while the entire simulation time (500 min) was not enough for the flow to reach 1 m depth in the old road type. The computed flow velocities at the highlighted points A, B, C, and D in the road section (see Fig. 3) are presented in Fig. 12. The Darcy flow velocity decreased with depth in both types of road. At the upper points, there was a clear jump in the velocity curve due to flux arrival. The flow velocity at points C and D was very low or approximately zero. Flow velocity was generally higher in the modern road; at points A and B (base and sub-base material) it was approximately 10^−4^ m/s, but in the old road type, it was less than 10^−5^ m/s for similar points beneath the road shoulder.

Most groundwater vulnerability assessments along roads (e.g., Gontier and Olofsson 2002) consider the importance of the surrounding geological material for the infiltration and spread of pollutants. However, if pollutants primarily infiltrate in the coarse road shoulder, e.g., due to traffic accidents, they will percolate downwards through the road embankment. Geological maps showing natural soils are therefore not always relevant for analysis of pollutant spread into roads, since calculations using natural soils usually give much smaller percolation velocities. However, geological maps are usually important for vulnerability assessment of soil and groundwater pollutants around the roads.

The calculated transport times based on resistivity measurements were of the same magnitude as the analytical calculations for sand and the dynamic 1D modeling results for the road shoulder and the inner ditch slope. Results from 2D COMSOL simulations for base and sub-base material gave reasonable transport times, but generally longer than analytical simulations and shorter than 1D modeling for both types of road. In reality, road constructions usually comprise several materials with great differences in hydraulic properties, e.g., a usually coarse embankment but often several layers building up the road verge (inner ditch slope) in order to meet different requirements regarding runoff, stability, and maintenance. The material in old roads is usually more similar to the surrounding geological materials than modern road constructions. Dynamic modeling is an excellent tool for vulnerability assessment of roads if the material hydraulic properties and the structure of the road are known. Tracer tests combined with resistivity measurements and 3D inverse modeling seem to be an excellent non-destructive method for identifying flow paths and transport times, but it lacks accuracy since every measurement gives a bulk value over a volume of the ground and, hence, an average value of the resistivity situation at the specific measurement time. However, due to the often large soil heterogeneity, preferential flows sometimes occur, which is not easily modeled and not clearly detected in the resistivity measurements.

## Conclusions

Tracer tests along roads in Sweden identified two major road types: modern roads built up of macadam with a highly permeable road shoulder and very short transport times, and older roads mainly consisting of natural and compacted soils, in this case gravelly-sandy till with much slower infiltration and percolation. All measured road stretches showed great hydraulic heterogeneity, often with channel flow. Field tests indicated that accidental spills of water-borne pollutants may infiltrate into the road shoulder and inner ditch, and the percolation time downwards in all tests was fairly rapid. Therefore, it is recommended that mitigation measures after traffic accidents commence within 0.5–1 h in areas vulnerable to pollution. Due to the large variation between test sites, investigations at specific vulnerable objects, such as water supply wells, should be carried out in advance. Vehicle fires in such vulnerable areas should not be treated using water-borne chemicals. Information on the road construction material and the hydraulic properties of the road shoulder should be obtained in advance at vulnerable sites, since natural soil maps are generally not sufficient for risk assessments.

The methodology using resistivity measurements and 3D inverse modeling can be a useful tool for tracing infiltration and percolation of water-borne substances. Comparing resistivity tracer tests in the road shoulder with 1D and 2D flow modeling showed results in the same order of magnitude. The advantage of using resistivity measurements is that such measurements are non-destructive, and that, representative flow patterns and flow velocities can be determined even in hydraulically heterogeneous environments.

Four different methods (dynamic flow modeling with 1D and 2D, analytical calculations, resistivity measurements, and modeling) indicated rapid transport processes in the road shoulder and inner ditch slope. Resistivity measurements can also provide a basis for analyzing the hydraulic properties of the roads, which can be used in dynamic modeling of pollutant spread from roads. The different methods used in this study complemented each other and can be applied to obtain site-specific information on conditions along vulnerable road stretches, which in turn can be used to devise specific measures necessary to remedy the effects of accidental pollution of groundwater or nearby surface waters.
